# Analysis of the association between lactotransferrin (LTF) gene polymorphism and dental caries

**DOI:** 10.1590/S1678-77572010000200011

**Published:** 2010

**Authors:** Luiza Foltran AZEVEDO, Giovana Daniela PECHARKI, João Armando BRANCHER, Carlos Alberto CORDEIRO, Kamilla Gabriella dos Santos MEDEIROS, Alessandra Armstrong ANTUNES, Eduardo Silva ARRUDA, Renata Iani WERNECK, Luciana Reis de AZEVEDO, Rui Fernando MAZUR, Samuel Jorge MOYSÉS, Simone Tetü MOYSÉS, Fábio Rueda FAUCZ, Paula Cristina TREVILATTO

**Affiliations:** 1 DDS, MS, Graduate student, Center for Health and Biological Sciences, Pontifical Catholic University of Paraná, Curitiba, PR, Brazil.; 2 Undergraduate student, Center for Health and Biological Sciences, Pontifical Catholic University of Paraná, Curitiba, PR, Brazil.; 3 PhD, Center for Health and Biological Sciences, Pontifical Catholic University of Paraná, Curitiba, PR, Brazil.

**Keywords:** Dental caries, ocurrence, LTF, Gene polymorphism, Exon 2

## Abstract

**Objective:**

The present study evaluated the association between lactotransferrin (LTF) gene polymorphism (exon 2, A/G, Lys/Arg) and dental caries.

**Material and Methods:**

A convenience sample of 110 individuals, 12 years old, was divided into: group 1, 48 individuals without caries experience (DMFT=0), and group 2, 62 subjects with caries experience (DMFT≥1). DNA was obtained from a mouthwash with 3% glucose solution, followed by a scrapping of the oral mucosa. After DNA purification, polymerase chain reaction (PCR), single strand conformation polymorphism (SSCP) was performed to access the study polymorphism. The LTF A/G (Lys/Arg) polymorphism had been previously reported as located in exon 1.

**Results:**

Allele 1 of the study polymorphism was associated with low DMFT index and showed a protective effect against caries experience (OR=0.16, IC=0.030.76, p=0.01).

**Conclusion:**

Lactotransferrin A/G (exon 2, Lys/Arg) polymorphism was associated with susceptibility to dental caries in 12-year-old students.

## INTRODUCTION

Dental caries is a multifactorial infectious disease, whose etiology is related to microbial^2^, diet^30^ and host aspects^15^. Cavities may appear whether cariogenic microorganisms and carbohydrates are present in a susceptible individual during a certain time in the mouth^7^. So far, researches have investigated several biological determinants, which can influence the biofilm cariogenicity^11,30^, like saliva flow and composition^8,27^. A constant salivary flow efficiently eliminates microorganisms from oral cavity. Thus, a reduced flow may easily take to microbial growth, followed by teeth deterioration^3,25^.

Saliva presents various innate or acquired defense factors capable of inhibiting bacterial invasion, growth and metabolism by different mechanisms, as bacterial adherence and streptococci acid production^9,25,27^. Some salivary proteins have an antibacterial effect, like lysozime, lactoperoxidase, immunoglobulins, aglutinines, mucins and lactotransferrin^28^. At the molecular level, there is a functional overlapping among several salivary proteins^9^.

Lactotransferrin (LTF) is a multifunctional metalloprotein, that belongs to the transferrin family, with 80 kDa and 690 amino acids^13,17^. Lactotransferrin is produced in several tissues and is present in diverse organism fluids, such as saliva, tears, semen, sweat, colostrum, milk and nasal secretion^13,23^. Polymorphonuclear leukocytes contain a great quantity of LTF, which is considered a cytokine that plays a role in the protection against several infections^17^. Its antibacterial action is attributed to the property of removing iron, depriving the microorganisms from their essential element^15^. Lactotransferrin can modulate dental biofilm aggregation and development, inhibiting *Streptococcus mutans* adhesion^5,16^. A significant association was found between decayed surfaces and salivary lactotransferrin concentration^21^.

The LTF gene is organized into 17 exons, with a size that varies from 23 to 35 kb among humans^23^. It is located in human chromosome 3, position 3p21^12^. Sequence variations in gene coding regions may lead to abnormal protein structure, causing an altered function^24^. If the frequency of the rarer allele is higher than 1% this process is termed polymorphism. A polymorphism (A/G) (rs 1126478), in the second exon of the LTF gene, is responsible for the substitution from an amino acid lysine (Lys) to an arginine (Arg) in position 29. A variant containing a Lys residue has been associated with an increased antibacterial activity against *S. mutans*^29^.

Studies concerning the analysis of the association between genetic polymorphisms in genes of the host response and dental caries are rare^10,22^. Thus, the aim of this study was to investigate the association between the polymorphism A/G (Lys/Arg) in the LTF gene and dental caries in 12-year-old students.

## MATERIAL AND METHODS

### Sample Selection

The sample was composed of 110 unrelated, 12-year-old, Caucasian students of both genders from a private school of Curitiba, PR, Brazil. The students were allocated to the study only if the parent/caregiver returned the informed consent form, according to norms of the Ethical Research Committee of the Center for Health and Biological Sciences of the Pontifical Catholic University of Paraná, according to the Resolution 96/96 of the Health National Council, register n. 104.

Students were excluded when smokers, using orthodontic appliances, taking chronic antiinflammatory and antibiotics in the last 6 months, or with history of any disease known to compromise immune function.

The students were diagnosed according to the decayed, missing and filled teeth index (DMFT). Teeth were considered decayed when presenting either cavities or white lesions. Individuals were divided into two groups, according to caries experience: Group 1: 48 students without caries experience (DMFT=0); Group 2: 62 individuals with caries experience (DMFT≥1).

### DNA Collection

The sampling of epithelial buccal cells was performed as previously described^26^. Briefly, the individuals undertook a mouthwash after 1min, containing 5 mL 3% glucose. Following mouthwash, a sterile wood spatula was used to scrape oral mucosa. The tip of the spatula was then shaken into the retained mouthwash solution. Buccal epithelial cells were pelleted by centrifugation at 2000 rpm for 10 min. The supernatant was discarded and the cell pellet resuspended in 1.300 mL of extraction buffer [10 mM Tris-HCl (pH 7.8), 5 mM EDTA, 0.5% SDS]^1^.

### Polymerase chain reaction (PCR) / Single strand conformation polymorphism (SSCP)

**Figure 1:**
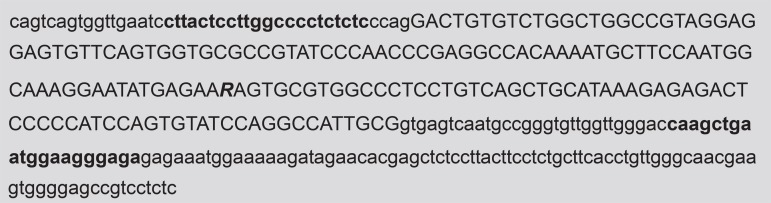
Lactotransferrin (LTF) gene sequence presenting the polymorphism A/G indicated by the letter ***R***. The capital letters refer to exon 2 (GenBank, NM_002343). Underlined boldface bases represent primers that flank the amplified sequence

For the PCR-SSCP 10.8 mL of final volume of reaction were prepared. One (1) mL of DNA was added to 9.4 mL of *PCR Supermix*^TM^ (Invitrogen Corporation, Carlsbad, USA) and 0.2 mL (5 *pmol*) of each primer. The following primers 5´ CTTACTCCTTGGCCCCTCTC 3´ (*forward*) e 5´ TCTCCCTTCCATTCAGCTTG 3´ (*reverse*) amplified a sequence of 238 base pairs (bp), located in the second exon of lactotransferrin gene. The study polymorphism was previously reported to be located in exon 1^29^. This study calls attention to the correct location of the study polymorphism, *exon 2* instead of *exon 1* (Figure 1).

The amplifications were carried out in a thermocycler Eppendorf Master Cycle Gradient (Eppendorf, Hamburg, Germany) and the reaction conditions were: initial denaturation at 96ºC for 3 min, followed by 40 cycles with denaturation at 94ºC for 30 s, annealing at 60ºC for 30 s and extension at 72ºC for 30 s, with a final extension of 10 min at 72ºC.

PCR amplified products were detected by means of SSCP, which consisted of DNA denaturation at 94ºC for 5 min, followed by fast maintenance on ice. The samples were placed on 7% polyacrylamide gel (39: 1), in which glycerol was added in a final concentration of 5%. The samples ran in 200 V, for a period of 5 h.

Gels were stained with silver nitrate, based on a described technique^20^. The SSCP technique does not allow identifying alleles. Only the sequencing can identify the alleles. In the case of this study, the alleles A and G were called 1 and 2.

### Statistical Analysis

The significance of the differences in the allelic and genotypic frequencies of the polymorphism studied between the groups with and without caries experience was accessed by chi-square test (Χ^2^) (*p* < 0.05).

## RESULTS

One hundred and ten (110) students with (n=62) and without (n=48) caries experience were analyzed. Of the students without dental caries experience (group 1), 19/48 individuals (39.6%) presented homozygous genotype (1/1), 22/48 students (45.8%) were heterozygote (1/2), and 7/48 (14.6%) had homozygous genotype (2/2). With regard to the group with caries experience (group 2), 16/62 individuals (25.8%) presented homozygous genotype 1/1, 30/62 students (48.4%) were heterozygote 1/2, and 16/62 (25.8%) were homozygote 2/2 (Table 1). The frequency of allele 1 in the group without caries experience was 62.5% (60/96) and of allele 2 was 37.5% (36/96). In the group with dental caries experience, the frequency of alleles 1 and 2 was of 50% (62/124) (Table 2). Although no statistically difference was found between the groups with and without dental caries experience in the genotypic frequency (*p*=0.19), a difference with a significance border line was realized in the allelic distribution (*p*=0.07) between the groups. Indeed, an association of allele 1 with low DMFT index was observed when individuals with lower DMFT values (≤ 2) were analyzed versus students with higher DMFT values (≥ 3) [1/1 + 1/2 vs 2/2, (OR=0.16, IC=0.03-0.76, *p*=0.01)]. Also, an association was found between allele 1 and increased levels of salivary flow (> 0.5 mL/min) (OR=2.48, IC=0.956.44, *p*=0.06).

**Table 1:** Distribution of genotypes in groups without (group 1) and with (group 2) caries experience

**Genotype**	**Group 1** ** n = 48 (%)**	**Group 2** ** n = 62 (%)**	***p value***
*1/1*	19 (39.6)	16 (25.8)	0.1939
*1/2*	22 (45.8)	30 (48.4)	
*2/2*	7 (14.6)	16 (25.8)	

**Table 2:** Allele frequencies in groups without (group 1) and with (group 2) caries experience

**Allele**	**Group 1**	**Group 2**	***p value***
	**n = 96 (%)**	**n = 124 (%)**	
*1*	60 (62.5)	62 (50)	0.0758
*2*	36 (37.5)	62 (50)	

## DISCUSSION

Saliva components and properties have been widely investigated^12^, but there are still some questions that need to be clarified. Results involving salivary proteins, such as LTF, are contradictory. Lactotransferrin presents bactericidal effect against some microorganisms and can modulate the aggregation and development of dental biofilm, inhibiting the adhesion of *S. mutans*^5,16^. Although LTF presents recognized antibacterial properties^17^, a low LTF expression was found in the saliva of subjects with reduced DMFT^9,21^, maybe by the fact that increasing in LTF production is due to an attempt to control the process of disease.

Polymorphisms in the LTF gene have been described^14,24^ and related with several pathologies, as Parkinson’s disease^6^, breast cancer^18^, leukemia^14^ and localized aggressive periodontitis^29^. However, apparently there are no studies investigating the association between polymorphisms in the LTF gene and dental caries.

Since functional genetic polymorphisms, that result in amino acid exchange, may affect protein function^19^, this study aimed to investigate the association between the polymorphism A/G (Lys/ Arg) in the LTF gene and dental caries in 12-yearold students by means of SSCP. Although the SSCP technique may not identify the alleles of polymorphic genes, it has the advantage of being a simple and rapid method to detect the presence of gene sequence variations. In this study, SSCP was used to search for variations in the second exon of LTF gene. The authors assumed that the variation observed in the migration pattern of amplified DNA fragments was due to the presence of an A to G exchange in LTF gene (exon 2), resulting in three possible genotypes (AA, AG and GG). Since the identification of allele A or G is not possible, the authors termed the alleles as 1 and 2.

It was not observed a statistically significant difference in the band migration pattern between the groups with and without caries experience in this population of 12-year-old students. However, a decrease in the frequency of allele 2 in the group without caries experience was found, which suggests that this allele could be contributing for the increasing in the susceptibility to dental caries. Thus, allele 1 could present a protective effect against caries experience, which was verified in this study. The polymorphism A/G in exon 2 results in an amino acid exchange (Lys for Arg). The variant presenting Lys residue shows an increased antibacterial activity against gram-positive microorganisms, including *S. mutans*^29^. Although the identification of the alleles is mandatory to realize which amino acid it brings, this study suggests the participation of one of the alleles in the disease status.

A previous study in the same population sample showed an association between salivary flow and dental caries experience^4^. In fact, a reduced salivary flow has been associated with higher caries experience, pointing to host factors in the determination of risk to dental caries in homogeneous populations, in terms of socioeconomic and environmental aspects. Patients with lower salivary flow were observed to show higher DMFT index and periodontal infection increase^11^. Thus, lower levels of caries were related with high salivary flow^9^. In this study, the same allele (allele 1) was associated with lower DMFT index and increased salivary flow, suggesting a protective effect in both saliva amount and quality.

To the best of our knowledge, this is the first study reporting the analysis of association between an LTF gene polymorphism and dental caries.

It was observed that allele 1 of polymorphism A/G, in exon 2 of the LTF gene, is associated with protection against dental caries experience in 12 years-old students. Additional studies with a higher number of individuals are necessary to confirm the association of the study polymorphism with susceptibility to dental caries. Moreover, other polymorphisms in different regions of LTF gene may also be responsible for alterations in the function of LTF protein. Sequences of DNA in the LTF gene promoter are special candidates for the association analysis of polymorphisms with the susceptibility to dental caries, where these sequences can be related to an increasing in the expression of the gene product. Besides, polymorphisms in genes that code for other salivary proteins with antibacterial function can also be contributing for the susceptibility to the dental caries and constitute potential targets for investigation. Moreover, although polymorphism studies of salivary proteins is scientifically intriguing, a polymorphism in only one of the defense proteins gene is not sufficient to explain a multifactorial disease as dental caries. There are several defense proteins in saliva, so that compensating mechanisms will be effective to overcome one small defect. Besides, oral hygiene, life style and diet, and saliva are so overwhelmingly important as factors related to dental caries, that a small ‘defect’ in LTF plays only a very minor role in maintaining the oral tissue healthy. Although LTF is a highly interesting antimicrobial compound, it is in saliva only a minor protein in the whole battery of antimicrobial mechanisms.

An understanding of the numerous salivary determinants at the molecular level has the potential to provide new ways of prevention and treatment of dental caries. Since caries is mostly diagnosed when cavities are present, the knowledge of risk at an individual level may provide more accurate cost-effective strategies for oral health promotion.

## CONCLUSION

Lactotransferrin A/G (exon 2, Lys/Arg) polymorphism was associated with susceptibility to dental caries in 12-year-old students. The study of other gene polymorphisms in LTF and in other salivary proteins may potentially contribute to the elucidation of the carious process.

## References

[1] Aidar M, Line SR (2007). A simple and cost-effective protocol for DNA isolation from buccal
epithelial cells. Braz Dent J.

[2] Ajdi D, Mcshan WM, Mclaughlin RE, Savi G, Chang J, Carson MB (2002). Genome sequence of Streptococcus Mutans UA159, a cariogenic dental
pathogen. Proc Natl Acad Sci.

[3] Atkison JC, Baum B (2001). Salivary enhancement: current status and future
therapies. J Dent Educ.

[4] Azevedo LF, Arruda ES, Santos TB, Brancher JA, Ignácio AS, Faucz FR (2005). Evaluation of socioeconomic aspects, salivary factors and oral habits
on the caries risk determination in 12-year-old students of a private school in
Curitiba, PR, Brazil. J Dent Clin Res.

[5] Berlutti F, Ajello M, Bosso P, Morea C, Andrea P, Giovanni A (2004). Both lactotransferrin and iron influence aggregation and biofilm
formation in Streptococcus mutans. Biometals.

[6] Borie C, Gasparini F, Verpillat P, Bonnet AM, Agid Y, Hetet G (2002). Association study between iron-related genes polymorphisms and
Parkinson's disease. J Neurol.

[7] Featherstone JDB (2004). The continuum of dental caries - evidence for a dynamic disease
process. J Dent Res.

[8] Gavião MBD, Bilt AV (2004). Salivary secretion and chewing: stimulatory effects from artifitial
and natural foods. J Appl Oral Sci.

[9] Jentsch H, Beetke E, Gocke R (2004). Salivary analyses and caries increment over 4 years an approach by
cluster analysis. Clin Oral Invest.

[10] Jonasson A, Eriksson C, Jenkinson HF, Källestål C, Johansson I, Strömberg N (2007). Innate immunity glycoprotein gp-340 variants may modulate human
susceptibility to dental caries. BMC Infect Dis.

[11] Kidd EAM, Fejerskov O What constitutes dental caries? Histopathology of carious enamel and
dentin related to the action of cariogenic biofilms. J Dent Res. 2004;83(sp. issue):35-8..

[12] Kim SI, Yu DY, Pak KW, Jeong S, Kim SW, Lee KK (1998). Structure of the human lactotransferrin gene and its chromosomal
localization. Mol Cells.

[13] Liu D, Wang X, Zhang Z, Teng CT (2003). An intronic alternative promoter of the human lactotransferrin gene is
activated by Ets. Biochem Biophys Res Commun.

[14] Liu LH, Gladwell W, Teng CT (2002). Detection of exon polymorphisms in the human lactotransferrin
gene. Biochem Cell Biol.

[15] Nariyama M, Shimizu K, Uematsu T, Maeda T (2004). Identification of chromosomes associated with dental caries
susceptibility using quantitative trait locus analysis in mice. Caries Res.

[16] Oho T, Mitoma M, Koga T (2002). Functional domain of bovine milk lactotransferrin which inhibits the
adherence of Streptococcus mutans cells to a salivary film. Infect Immun.

[17] Orsi N (2004). The antimicrobial activity of lactotransferrin: current status and
perspectives. Biometals.

[18] Penco S, Caligo MA, Cipollini G, Bevilacqua G, Garre C (1999). Lactotransferrin expression in human breast cancer. Cancer Biochem Biophys.

[19] Pociot F, Molvig J, Wogensen L, Worsaae H, Nerup J (1992). A TaqI polymorphism in the human interleukin-1 beta (IL-1 beta) gene
correlates with IL-1 beta secretion in vitro. Eur J Clin Invest.

[20] Sammons DW, Adams LD, Nishizawa EE (1981). Silver stainig in PAGE. Electrophoresis.

[21] Sikorska MH, Mielnik-Blaszczak M, Kapec E (2002). The relationship between the levels of SigA, lactotransferrin and
alpha (1) proteinase inhibitor in saliva and permanent dentition caries in
15-year-olds. Oral Microbiol Immunol.

[22] Slayton RL, Cooper ME, Marazita ML (2005). Tuftelin, mutans streptococci, and dental caries
susceptibility. J Dent Res.

[23] Teng CT (2002). Lactotransferrin gene expression and regulation: an
overview. Biochem Cell Biol.

[24] Teng CT, Gladwell W (2006). Single nucleotide polymorphisms (SNPs) in human lactoferrin
gene. Biochem Cell Biol.

[25] Tenovuo J (2002). Clinical applications of antimicrobial host proteins lactoperoxidase,
lysozyme and lactotransferrin in xerostomia: efficacy and safety. Oral Disease.

[26] Trevilatto PC, Line SR (2000). Use of buccal epithelial cells for PCR amplification of large DNA
fragments. Forensic Odontostomatol.

[27] Van Nieuw Amerongen A, Veerman EC (2002). Saliva -the defender of oral cavity. Oral Disease.

[28] Van Nieuw Amerongen A, Bolscher JGM, Veerman ECI (2004). Salivary proteins: protective and diagnostic value in
cariology?. Caries Res.

[29] Velliyagounder K, Kaplan JB, Furgang D, Legarda D, Diamondd G, Parkin RE (2003). One of two human lactotransferrin variants exhibits increased
antibacterial and transcriptional activation activities and is associated with
localized juvenile periodontitis. Infect Immun.

[30] Zero DT (2004). Sugars - the arch criminal. Caries Res.

